# Detection and localization of citrus fruit based on improved You Only Look Once v5s and binocular vision in the orchard

**DOI:** 10.3389/fpls.2022.972445

**Published:** 2022-07-29

**Authors:** Chaojun Hou, Xiaodi Zhang, Yu Tang, Jiajun Zhuang, Zhiping Tan, Huasheng Huang, Weilin Chen, Sheng Wei, Yong He, Shaoming Luo

**Affiliations:** ^1^Academy of Contemporary Agriculture Engineering Innovations, Zhongkai University of Agriculture and Engineering, Guangzhou, China; ^2^Academy of Interdisciplinary Studies, Guangdong Polytechnic Normal University, Guangzhou, China; ^3^School of Mechatronics Engineering and Automation, Foshan University, Foshan, China; ^4^Engineering Research Center for Intelligent Robotics, Jihua Laboratory, Foshan, China; ^5^College of Biosystems Engineering and Food Science, Zhejiang University, Hangzhou, China

**Keywords:** citrus detection, citrus localization, binocular vision, YOLO v5s, loss function

## Abstract

Intelligent detection and localization of mature citrus fruits is a critical challenge in developing an automatic harvesting robot. Variable illumination conditions and different occlusion states are some of the essential issues that must be addressed for the accurate detection and localization of citrus in the orchard environment. In this paper, a novel method for the detection and localization of mature citrus using improved You Only Look Once (YOLO) v5s with binocular vision is proposed. First, a new loss function (polarity binary cross-entropy with logit loss) for YOLO v5s is designed to calculate the loss value of class probability and objectness score, so that a large penalty for false and missing detection is applied during the training process. Second, to recover the missing depth information caused by randomly overlapping background participants, Cr-Cb chromatic mapping, the Otsu thresholding algorithm, and morphological processing are successively used to extract the complete shape of the citrus, and the kriging method is applied to obtain the best linear unbiased estimator for the missing depth value. Finally, the citrus spatial position and posture information are obtained according to the camera imaging model and the geometric features of the citrus. The experimental results show that the recall rates of citrus detection under non-uniform illumination conditions, weak illumination, and well illumination are 99.55%, 98.47%, and 98.48%, respectively, approximately 2–9% higher than those of the original YOLO v5s network. The average error of the distance between the citrus fruit and the camera is 3.98 mm, and the average errors of the citrus diameters in the 3D direction are less than 2.75 mm. The average detection time per frame is 78.96 ms. The results indicate that our method can detect and localize citrus fruits in the complex environment of orchards with high accuracy and speed. Our dataset and codes are available at https://github.com/AshesBen/citrus-detection-localization.

## Introduction

Citrus plays an essential role in the fruit industry around the world, with an annual production of approximately 140 million tons (Zheng et al., 2021; Noorizadeh et al., 2022). As the cost of fruit harvesting increases and the availability of skilled labor decreases in China, the traditional manual harvesting method is no longer practical (Gongal et al., 2015; Tang et al., 2021). Presently, fruit harvesting has become increasingly automated for labor-saving and large-scale agriculture (Onishi et al., 2019). The development of an automated citrus picking robot is an inevitable trend for fruit harvesting (Zhuang et al., 2018). In recent work, the development of automatic fruit picking with a robot involves two main tasks: (1) fruit detection and (2) fruit localization via computer vision. The accuracy of fruit detection and fruit localization directly determines the picking efficiency of the robot.

Fruit detection using computer vision has been investigated in numerous recent studies, and most have applied deep learning methods to achieve good performance and robustness (Yang et al., 2020; Chen et al., 2021; Yan et al., 2021). Wan and Goudos (2020) integrated multiclass classification into Faster R-CNN to detect oranges, apples, and mangoes. The improved model achieved a 90.72% mAP. Kang and Chen (2020) proposed a LedNet network with a feature pyramid network and an atrial space pyramid pool for mature apple detection; the recall rate and precision were 0.821 and 0.853, respectively. Chu et al. (2021) improved mask R-CNN by adopting a suppression branch to suppress the generation of nonapple fruit features. However, their method has poor detection performance under backlight conditions. He et al. (2020) developed a deep bounding box regression forest to describe the characteristics of immature citrus on three levels, which is beneficial for differentiating an object from the background. However, the detection speed is slow (0.759 s per frame), making it challenging to apply in real-time applications. For the real-time application of fruit harvesting, the detection speed should be at least 10–15 frames per second (Tu et al., 2020). YOLO series models have been used in various applications for fast detection speed with high accuracy (Jiang et al., 2020; Wang et al., 2021). Xiong et al. (2020) used a YOLO v2 model to detect green mango and reported a recall of 89.0%, a precision of 96.1%, and an average detection time of 0.08 s per frame. Liang et al. (2020) combined YOLO v3 and U-Net to detect litchi fruits and litchi stems at night for picking robots under different illuminations; 96.1% precision and 89.0% recall were achieved. However, the method has not yet been assessed in the daytime. Wang and He (2021) developed an improved YOLO v5 model to detect apple fruitlets using the channel pruning method. However, the network architecture must be manually adjusted during detection. Notably, the target-background class imbalance is typically the main obstacle encountered in training convolutional neural networks (Buda et al., 2018). To address such class imbalance, Lin et al. (2020) designed a focal loss function to make the network pay more attention to hard samples in training, but the approach cannot push the object further from the background. Rahman et al. (2020) proposed polarity loss to improve focal loss. In the above studies, various deep learning methods have been proposed to detect fruit targets and have achieved good results. However, the detection performance deteriorates in unstructured growing environments with variable illumination conditions. For better accuracy, the disparity between citrus and background under variable illumination conditions and different occlusion states should be incorporated into the network structure.

The purpose of fruit localization is to determine the spatial coordinates of the detected fruit and its location information, such as posture and shape (Huang et al., 2019). Many fruit localization methods require a binocular stereo vision system. The depth map or point cloud image is captured to obtain three-dimensional (3D) localization of fruit. Yang et al. (2020) employed a mask R-CNN to detect citrus objects and branches and matched the color and depth maps to locate fruits and branches. The average error in the diameter of the fruit and the branch was less than 4 mm. However, the distance from the fruit to the camera was not provided in their work. Nguyen et al. (2016) used a Euclidean clustering algorithm to segment a single apple using a point cloud image. The results showed that the errors in the spatial coordinates and the diameter of the fruit were slightly less than 10 mm, but the 3D location information about apples was not the aim of their work. Xu et al. (2018) proposed the PointFusion structure to estimate the 3D object bounding box and its confidence from RGB image and point cloud information. The approach produces good results in the KITTI and SUN-RGBD datasets, with 78% AP. Since the information of the depth map or point cloud is incomplete, fruit localization often requires the use of empirical knowledge (Liu et al., 2017). Wang et al. (2017) adopted Otsu’s method and a one-dimensional filter to remove occluded objects (leaves, branches, fruit particles, etc.) and employed ellipse fitting to extract a well-separated mango region. Finally, mango dimensions were calculated using depth information. Ge et al. (2020) developed a shape completion method to reconstruct the point clouds of strawberries; the average error of the center point of strawberries was 5.7 mm. However, the reconstructed error is larger in the case of the neighboring overlapping fruits. Note that an incomplete depth map makes it difficult to recover the missing depth value lost by variable illumination or the fruit region being occluded by randomly overlapping participants, such as neighboring fruits and other background objects. Therefore, this paper aims to restore the depth map with high accuracy for locating fruits in unstructured orchard environments.

The objective of this work is to develop a novel method for the detection and localization of mature citrus fruits in natural orchards using a binocular camera. The pipelines of the study are to (1) design a new loss function to enhance the detection performance of the YOLO v5s network architecture under variable illumination conditions, (2) extract the fruit region in the RGB image and recover the missing value in the depth map under different occlusion states of citrus fruit, and (3) estimate the 3D localization of citrus fruits using the camera imaging model and the geometric features of citrus fruits. Our method can provide 3D localization information of citrus fruits, such as the diameters of citrus fruits in the 3D direction, the spatial coordinates of citrus fruits, the distance between citrus fruits and the camera, and the 3D bounding box of citrus fruits.

## Materials and methods

### Datasets

A variety of citrus named “Shantanju” was investigated in the hillside orchard of the Guangzhou Conghua Hualong Fruit and Vegetable Freshness Co. Ltd., located in Guangzhou, China (113°39’2.38’E, 23°33’12.48’N). A total of 4855 groups of images were captured in December 2020 and December 2021 before harvest. Image acquisition was performed using a binocular camera (Model ZED 2, Stereolab’s Co. Ltd, USA) with a 1920 × 1080 pixel resolution under sunny and cloudy conditions. The distance between the camera and citrus was set to approximately 30∼150 cm. Each group of images contains a left view (RGB image) and a depth map (grayscale image). Note that the right view images were also captured and used only to generate the depth map with the left view images. The depth map is provided with a Z value for every pixel (X, Y) in the left view image. According to the illumination of the citrus surface, images are divided into three groups: non-uniform illumination (non), weak illumination (weak), and well illumination (well). In total, 2913 images were randomly selected as the training dataset (train), 971 images were selected as the validation dataset (validation), and 971 images were selected as the test dataset (test), the number of citrus samples in each group is shown in Table 1.

**TABLE 1:** Dataset distribution.

	Non		Weak		Well		Total	
	Images	Samples	Images	Samples	Images	Samples	Images	Samples
Train	923	4569	814	2503	1176	4016	2913	11088
Validation	333	1636	255	809	383	1435	971	3880
Test	307	892	269	655	395	1052	971	2599

**FIGURE 1:**
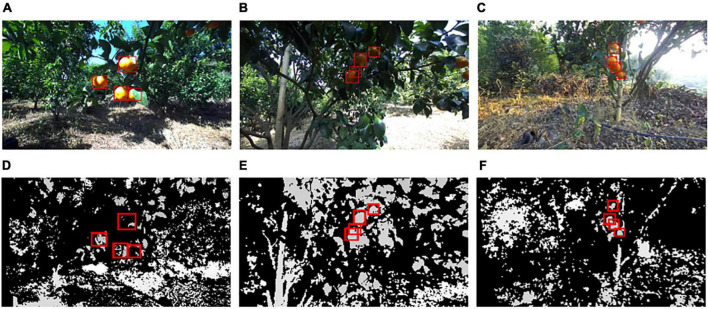
Examples of citrus images captured in three illumination conditions: **(A)** non, **(B)** weak, **(C)** well, **(D)** depth map of **(A)**, **(E)** depth map of **(B)**, and **(F)** depth map of **(C)**.

The hand of the harvesting robot is designed to pick citrus fruits that are in the correct position in front of the camera. In each left view image, the citrus fruits located near the center of the image were manually labeled with bounding boxes using Labelme software. Figures 1A–C provides examples of citrus images from each illumination group. The bounding boxes of labeled citrus are annotated with red rectangles. The corresponding depth maps with labeled citrus are shown in Figures 1D–F, where the grayscale of color is based on distance from the camera, i.e., closer objects are darker; further objects are lighter.

### Detection and localization of citrus

**FIGURE 2:**
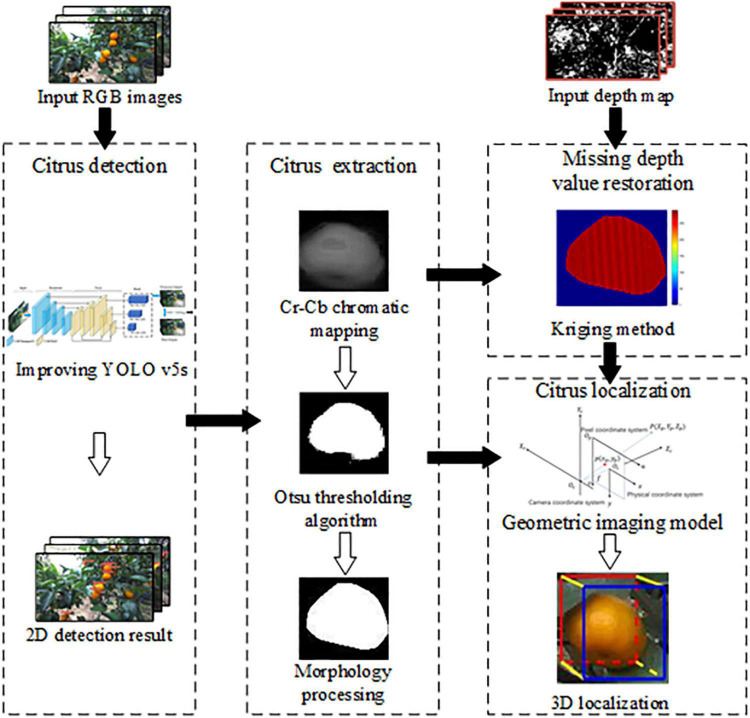
Flow diagram of our proposed method.

An overview of our proposed method for citrus detection and localization is presented in Figure 2. The main procedure involves the following steps: Firstly, an improved YOLO v5s is developed to detect citrus in the 2D bounding box. Secondly, Cr-Cb chromatic mapping, Otsu threshold algorithm, and morphology processing are used to extract citrus shape. The missing depth values are recovered by the kriging method. Finally, the 3D localization of citrus fruit is realized by geometric imaging model. Each procedure is described in detail in the following subsections.

#### Detection of the 2D bounding box of citrus fruit

**FIGURE 3:**
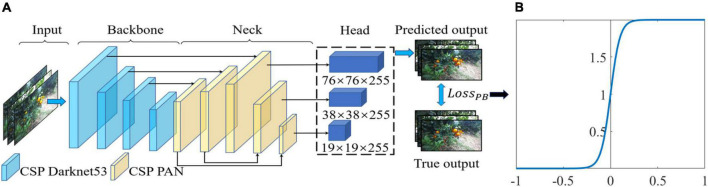
Citrus detection model based on You Only Look Once (YOLO) v5s: **(A)** network structure of improved YOLO v5s and **(B)** function graph of penalty function *f*_*p*_.

YOLO (You Only Look Once) is a one-stage detection network that converts object detection into a regression problem using convolutional neural networks (Wang et al., 2021, 2022). YOLO v5, the latest version of the YOLO model (Jocher and Stoken, 2021), has a faster detection speed and higher accuracy than the previous version. The release of YOLO v5 consists of four different model sizes: YOLO v5s (smallest), YOLO v5m, YOLOv5l, and YOLO v5x (largest). The network structures of these four models are basically the same, but the numbers of modules and convolution kernels are different. Considering that the application scenario of this paper requires fast detection efficiency, the YOLO v5s model is selected as the basic network, and its structure is shown in Figure 3A. The YOLO v5s network is divided into three parts. The first part is the backbone network, which is responsible for the feature extraction of the target. The second part is PANet, which generates feature pyramids for object scaling. The third part is the head network, which conducts the final detection.

In YOLO v5s, binary cross-entropy with a logit loss function (*Loss*_*B*_) is used to calculate the class probability and objectness score for each sample, as follows:


(1) $$\begin{array}{c} L\,o\,s\,s_{B}\,\left(x_{i},y_{i}\right)=-y_{i}\,\log\left(\sigma \,\left(x_{i}\right)\right)-\left(1-y_{i}\right)\,\log\left(1-\sigma \,\left(x_{i}\right)\right) \end{array},$$

where *i* is the sample index, *x*_*i*_ is the predicted likelihood, *y*_*i*_ stands for the ground truth, and σ(⋅) is the sigmoid function that maps the prediction *x*_*i*_ to the probability for the ground truth. In object detection tasks, the problem of unbalanced training sets is considerable (Lin et al., 2020), i.e., the background information in the dataset used for training is overrepresented compared to that of the target class. The sum of *Loss*_*B*_ from the easy samples over the entire images can overwhelm the overall *Loss*_*B*_ from the hard samples. Moreover, the training is inefficient, as most locations are easy samples that do not contribute to learning. Furthermore, in our trial-and-error experiments, the hard negative samples, i.e., the citrus misclassified as background, are difficult to distinguish from the background under weak illumination or obvious occlusion. On the other hand, the hard positive samples, i.e., the background misclassified as a citrus target, exhibit similar characteristics to mature citrus due to the uncontrolled factors in the orchard environment.

To better differentiate citrus from the background under variable illumination conditions and different occlusion states, we design a new loss function, the polarity binary cross-entropy with logit loss (*Loss*_*PB*_), to calculate the class probability and objectness score to penalize the hard samples. In particular, a penalty function *f*_*p*_ (Rahman et al., 2020) is developed to represent the disparity between the prediction for citrus and background. *Loss*_*PB*_ is defined as follows:


(2) $$\left\{ \begin{array}{c} L\,o\,s\,s_{P\,B}\,\left(x_{i},y_{i}\right)=f_{p}\,\left(\sigma \,\left(x_{i}\right)\right)\,L\,o\,s\,s_{B}\,\left(x_{i},y_{i}\right)\\ f_{p}\,\left(z_{i}\right)=\frac{2}{1+\exp\left(-\gamma \,\left({\bar{z}}_{i}-z_{i}\right)\right)} \end{array}\right.$$

where *z*_*i*_ is the probability of sample *i* being predicted as the true class, such as citrus target or background, ${\bar{z}}_{i}=1-z_{i}$ is the probability of sample *i* being misclassified as the incorrect class, and γ is a slope parameter of the sigmoid function *f*_*p*_ (Figure 3B). *f*_*p*_ is used to calculate the disparity between the prediction for the true class and false class based on the value of ${\bar{z}}_{i}-z_{i}$. If the citrus target is misclassified as background, the prediction probability ${\bar{z}}_{i}$ is greater than, such that a large value of ${\bar{z}}_{i}-z_{i}$ is obtained, and a large penalty will be assigned by *f*_*p*_. In this case, the penalty value of *Loss*_*PB*_ is larger than that of *Loss*_*B*_, which helps to suppress the missed detection of citrus. Similarly, if the background is misclassified as citrus, a large penalty will be assigned by *f*_*p*_ due to the large value of ${\bar{z}}_{i}-z_{i}$, which will improve the false detection of citrus. On the other hand, if a citrus target or the background is predicted with a more reliable probability of *z*_*i*_, the penalty value applied by *f*_*p*_ will be closer to 0 due to the small value of ${\bar{z}}_{i}-z_{i}$. In such a case, the penalty value of *Loss*_*PB*_ is smaller than that of *Loss*_*B*_ and is pushed toward zero. In general, a large penalty is applied to missed detection and false detection of citrus targets. Thus, *f*_*p*_ enforces a large margin to push predictions *z*_*i*_ and ${\bar{z}}_{i}$ further apart.

Recall rate (*R*), precision (*P*), and *F*_β_-score (*F*_β_) are selected to evaluate the performance of the improved YOLO v5s in the test dataset:


(3) $$R=\frac{T\,P}{T\,P+F\,N},$$

(4) $$P=\frac{T\,P}{T\,P+F\,P},$$

(5) $$F_{\beta }=\left(1+\beta^{2}\right)\,\frac{P\times R}{\beta^{2}\times P+R},$$

where *FN* is the number of false negatives for the false detection of citrus samples, *FP* is the number of false positives for the missed detection of citrus samples, and *TP* is the number of true positives for the detected citrus samples. *F*_β_ uses a positive real number β to weigh the importance between *R* and *P*. In this paper, β is set to 1 as *F*_*1*_ by regarding *R* and *P* are equally necessary for our experiment.

#### Extraction of the citrus fruit region from the 2D bounding box

Image data captured in a natural orchard always contain multiple participants, e.g., grass, soil, lawn, leaves, branches, trunks, and sky. The citrus fruit region is difficult to extract exactly from the 2D bounding box predicted from the improved YOLO v5s. Fortunately, these participants have different color characteristics, so the different targets can be extracted based on their color information. Here, the proper color space is beneficial to robustly extract the citrus fruit region from the background. Zhuang et al. (2018) and Zhuang et al. (2019) adopted improved R-G chromatic mapping to extract fruit regions. In this paper, the input images are converted into the YCbCr color space for better contrast enhancement between the citrus fruit region and background.

**FIGURE 4:**
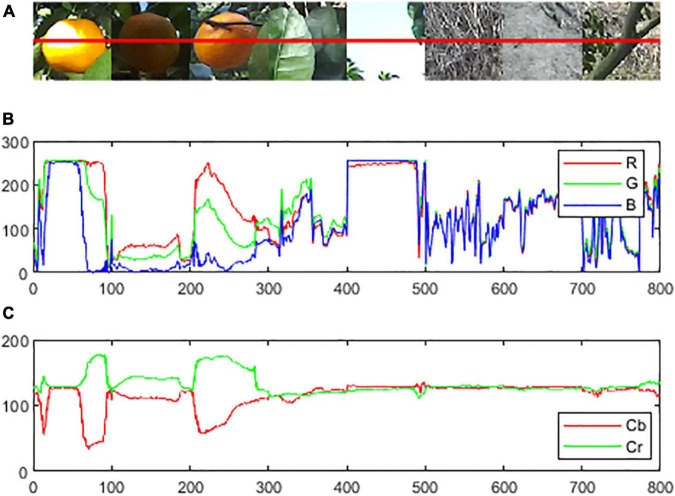
Examples of color curves of the citrus and background in different color spaces: **(A)** original RGB image, **(B)** color intensity on the line on R, G, and B elements in RGB color space, and **(C)** color intensity on the line on Cb and Cr elements in YCrCb color space.

As shown in Figure 4A, a horizontal red line was drawn across citrus fruits and the background. The color intensities of the pixels of the line are represented with the R curve (the red element of RGB), the G curve (the green element), and the B curve (the blue element) in Figure 4B. The Cr curve (the Cr element of YCbCr) and the Cb curve (the Cb element) are represented in Figure 4C. The intensity difference between the R curve and G curve is small in both the citrus region and background, and there are no obvious rules exhibited in the B curve among the citrus fruit regions and backgrounds. However, the intensity difference between the Cr curve and Cb curve values within the citrus region is obviously greater than that of the background. Thus, Cr-Cb chromatic mapping is suitable to enhance the disparity between the citrus region and the background participants.

**FIGURE 5:**
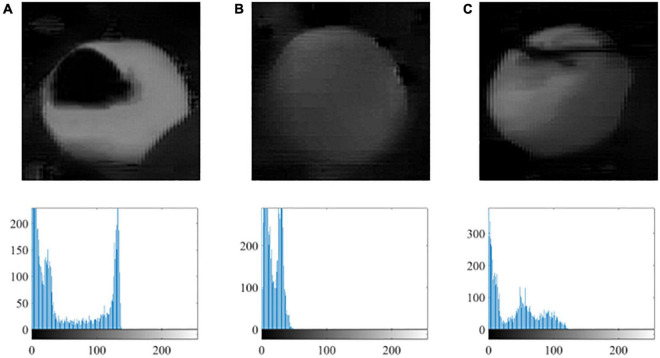
Examples of the citrus image after Cr-Cb chromatic mapping and its gray histogram under variable illumination: **(A)** non, **(B)** weak, and **(C)** well.

The Otsu thresholding algorithm is an appropriate method to segment the potential citrus regions from the background, where the best threshold value is selected by maximizing the variance between foreground and background. As shown in Figure 5, the Cr-Cb chromatic mapping has prominent bimodal characteristics in the intensity histogram under variable illumination, where the citrus fruit region contributes to the high value and background contributes to the lower value. Therefore, the best threshold value from Otsu is suitable to segment the citrus fruit region from the background.

The fruit region segmented by Otsu thresholding will not be complete in terms of shape due to the irregular growth situations of citrus fruit that are occluded by adjacent fruits, branches and leaves. To address this problem, the mathematical morphology operations of erosion, dilation, and hole filling are subsequently adopted to fill the gaps between detected regions, remove noise, fill small holes, and smooth the region’s boundary. Then, the mathematical morphology operation of convex hull is used to estimate the occluded regions of the fruit from the partially compact region. In this way, the citrus fruit can be almost completely segmented from its corresponding 2D bounding box.

#### Recovery of missing depth values

To achieve the 3D localization result of citrus, it is essential to obtain a complete depth map of the whole citrus fruit region; however, an incomplete depth map is always obtained for two main reasons. First, the depth map is sparse in the case of binocular stereo conditions. The depth value is missing and set to zero for pixels where no depth information is sensed by the ZED camera, which may be caused by variable illumination, camera performance limitation, and shooting angle (Liu et al., 2017). Second, the depth values can be missing due to the occluded region estimated from the morphological processing. To restore the complete depth map of the citrus region, the kriging method is adopted to predict the missing depth value by adding the weight of the observed depth value.

Let *I*_*O*_ be the citrus region segmented by Otsu thresholding and *I*_*C*_ be the citrus fruit region extracted via the convex hull operation. We denote by *I*_*in*_ the set of pixels whose depth value is missing in *I*_*C*_, such that the depth value is zero or the pixel is located outside of *I*_*O*_. Let *I*_*V*_ be the set of pixels whose observed depth value is available in *I*_*O*_. Therefore, the missing depth value in *I*_*in*_ can be obtained as follows:


(6) $$\overset{\wedge }{Z}\left(s\right)=\sum_{p\in I_{V}}\lambda_{p}\,\left(s\right)\,Z\,\left(p\right),\forall \text{s}\in {\text{I}}_{i\,n},$$

where *Z*(*p*) is the observed depth value at pixel *p* and λ_*p*_(*s*) is the weight of *Z*(*p*), which depends not only on the distance between the depth values but also on the position and overall spatial arrangement of the observed depth value around pixel *s*. Note that the kriging method is the best linear unbiased estimator to restore the missing depth value using observed depth values from the incomplete depth map. Therefore, all the missing depth values in *I*_*C*_ will be restored completely.

#### 3D localization of citrus fruit

The 3D localization of citrus determines the spatial position and posture information, such as citrus diameter in the 3D direction *d*_*x*_, *d*_*y*_, and *d*_*z*_, the spatial coordinates of citrus *Q*_0_(*X*_*q*_,*Y*_*q*_,*Z*_*q*_), the distance between the citrus and camera *d*, the spatial coordinates of the citrus 3D bounding box *P*_1_,*P*_2_,…,*P*_8_, and the corresponding 2D coordinates of the 3D bounding box in the image plane *p*_1_,*p*_2_,…,*p*_8_. The 3D coordinates of a point in the real world must be precisely mapped to the 2D coordinates of a pixel in the imaging plane. Here, the transformation relation among the camera coordinate system *O*_*c*_, the physical coordinate system *O*_*i*_, and the pixel coordinate system *O*_*p*_ should be analyzed.

**FIGURE 6:**
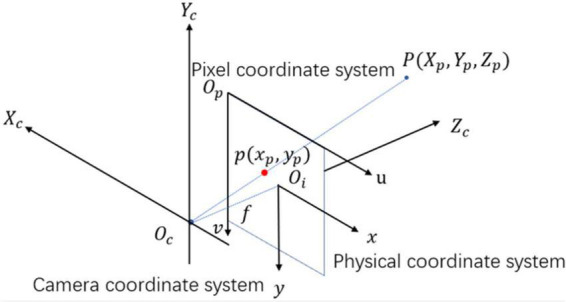
Coordinate system transformation diagram.


As illustrated in Figure 6, a physical coordinate system *O*_*i*_ is depicted with the origin in the imaging plane (unit: millimeter). The camera coordinate system *O*_*c*_ is created with the optical center as the coordinate origin. Note that the coordinates of the object in the real world are represented relative to *O*_*c*_, and *O*_*c*_ reaches *O*_*i*_ through perspective projection transformation. Suppose the coordinates of point *P* in *O*_*c*_ are (*X*_*p*_,*Y*_*p*_,*Z*_*p*_), and the corresponding coordinates projected onto *O*_*i*_ are (*x*_*p*_,*y*_*p*_). The relationship of point *P* between *O*_*c*_ and *O*_*i*_ is given by

(7) $$\left\{ \begin{array}{c} x_{p}=f\,\frac{X_{p}}{Z_{p}}\\ y_{p}=f\,\frac{Y_{p}}{Z_{p}} \end{array}\right.$$

where *f* is the camera focal length. As demonstrated in Figure 6, a pixel coordinate system *O*_*p*_ is depicted with the origin on the top-left vertex of the image (unit: pixel). The *u-* and *v-*axes are parallel to the *x-* and *y-*axes of *O*_*i*_. Let the point (*u*_*p*_,*v*_*p*_) in *O*_*p*_ corresponding to the point (*x*_*p*_,*y*_*p*_) in *O*_*i*_. The two coordinate values can be obtained as follows:


(8) $$\left\{ \begin{array}{c} u_{p}=\frac{x_{p}}{d_{u}}+u_{0}\\ v_{p}=\frac{y_{p}}{d_{v}}+v_{0} \end{array}\right.$$

where (*u*_0_,*v*_0_) represents the translation of the origin of *O*_*i*_ relative to the origin of *O*_*p*_ and *d*_*u*_ and *d*_*v*_ represent the actual size of the pixels in the *u-*axis and *v-*axis directions, respectively. According to Eqn. (7) and (8), the transformed relationship between *O*_*p*_ and *O*_*c*_ is given as


(9) $$Z_{p}\,\left[\begin{array}{c} u_{p}\\ v_{p}\\ 1 \end{array}\right]=\left[\begin{array}{ccc} f_{x} & 0 & u_{0}\\ 0 & f_{y} & v_{0}\\ 0 & 0 & 1 \end{array}\right]\,\left[\begin{array}{c} X_{p}\\ Y_{p}\\ Z_{p} \end{array}\right],$$

where *f*_*x*_ = *f*/*d*_*u*_, *f*_*y*_ = *f*/*d*_*v*_. Note that *f*, *d*_*u*_, *d*_*v*_, *u*_*0*_, and *v*_*0*_ are the intrinsic camera parameters that can be provided from the factory parameters of the ZED camera, and *Z*_*p*_ is the observed depth value of the depth map.

**FIGURE 7:**
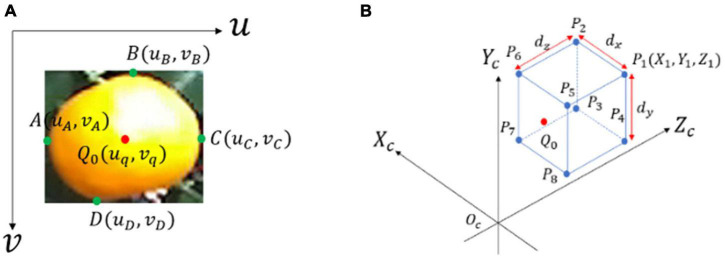
Example of 2D and 3D location information of citrus fruit: **(A)** 2D information of citrus fruit with four endpoints (green points) and center points *Q*_*0*_ (red points) in *O*_*p*_. **(B)** Citrus 3D bounding box in *O*_*c*_ with eight vertices.


As shown in Figure 7A, let A, B, C, and D be the leftmost, topmost, rightmost, and bottom-most endpoints of the citrus fruit region projected in *O*_*i*_, respectively, which have coordinates (*u*_*A*_,*v*_*A*_), (*u*_*B*_,*v*_*B*_), (*u*_*C*_,*v*_*C*_), and (*u*_*D*_,*v*_*D*_). Denote (*X*_*A*_,*Y*_*A*_,*Z*_*A*_), (*X*_*B*_,*Y*_*B*_,*Z*_*B*_), (*X*_*C*_,*Y*_*C*_,*Z*_*C*_), and (*X*_*D*_,*Y*_*D*_,*Z*_*D*_) as the corresponding spatial coordinates of points A, B, C, and D in *O*_*c*_. According to Eqn. (9), the spatial coordinates of A, B, C, and D are given by

(10) $$\left[\begin{array}{c} X_{i}\\ Y_{i} \end{array}\right]=\left[\begin{array}{ccc} \begin{array}{c} f_{x}\\ 0 \end{array} & \begin{array}{c} 0\\ f_{y} \end{array} & \begin{array}{c} u_{0}\\ v_{0} \end{array} \end{array}\right]\,\left[\begin{array}{c} u_{i}\\ v_{i}\\ Z_{i} \end{array}\right],$$

where *i* is A, B, C and D. Let *d*_*x*_, *d*_*y*_ and *d*_*z*_ be the fruit diameter in the *X*_*c*_-, *Y*_*c*_-, and *Z*_*c*_-axes, respectively. *d*_*x*_ and *d*_*y*_ are obtained according to the spatial coordinates of A, B, C, and D,


(11) $$\left\{ \begin{array}{c} d_{x}=X_{C}-X_{A}\\ d_{y}=Y_{D}-Y_{B} \end{array}\right.$$

In 3D perspective projection, the citrus fruit diameter *d*_*z*_ cannot be obtained directly from the image. Fortunately, the shape of a citrus fruit is similar to an ellipsoid; thus, the magnitudes of *d*_*x*_, *d*_*y*_, and *d*_*z*_ will be highly correlated. In this paper, *d*_*z*_ can be estimated by fitting a quadratic polynomial function of *d*_*x*_ and *d*_*y*_:


(12) $${\hat{d}}_{z}=\beta_{0}+\beta_{1}\,d_{x}^{2}+\beta_{2}\,d_{y}^{2}+\beta_{3}\,d_{x}+\beta_{4}\,d_{y},$$

where β_0_,β_1_,…,β_4_ are the regression coefficients of a polynomial that can be determined using the least-squares method.

Let *Q*_0_(*u*_*q*_,*v*_*q*_) be the center point of the citrus 2D bounding box (Figure 7), which indeed corresponds to the center of the citrus surface. The spatial coordinates (*X*_*q*_,*Y*_*q*_,*Z*_*q*_) of *Q*_*0*_ in *O*_*c*_ are obtained using Eqn. (10). Denote by *d* the Euclidean distance from *Q*_*0*_ to the origin point, i.e., the distance between the citrus and camera,


(13) $$d=\sqrt{X_{q}^{2}+Y_{q}^{2}+Z_{q}^{2}}.$$

The position and posture information for detected targets can usually be determined by the 3D bounding box (Xu et al., 2018). Let *P*_1_,*P*_2_,…,*P*_8_ be the eight vertices of the citrus 3D bounding box (Figure 7B), which have coordinates of (*X*_*i*_,*Y*_*i*_,*Z*_*i*_) for *i* = 1, 2,…, 8. In particular, (*X*_*i*_,*Y*_*i*_,*Z*_*i*_) can be obtained from the relative geometrical position of *P*_*i*_ to *Q*_*0*_, e.g., (*X*_1_,*Y*_1_,*Z*_1_) is inferred as follows:


(14) $$\left\{ \begin{array}{c} X_{1}=X_{q}-d_{x}\,/\,2\\ Y_{1}=Y_{q}+d_{y}\,/\,2\\ Z_{1}=Z_{q}+d_{z} \end{array}\right.$$

To visualize the 3D bounding box of citrus in the image, the corresponding projected 2D coordinates are calculated. Let the eight vertex points *p*_1_,*p*_2_,...,*p*_8_ be the corresponding *P*_1_,*P*_2_,…,*P*_8_ projected on *O*_*p*_, which have coordinates (*u*_*i*_,*v*_*i*_), *i* = 1, 2,…, 8. They can be deduced by Eqn. (9). Therefore, the 3D localization for each citrus is summarized in Algorithm 1.

**Table:** Algorithm 1 - Calculation of 3D localization for a citrus fruit.

**Input:** Citrus fruit region *I*_*C*_ and depth map *I*_*d*_. **Output:***d*_*x*_, *d*_*y*_, *d*_*z*_, *Q*_0_(*X*_*q*_,*Y*_*q*_,*Z*_*q*_), *d*, (*u*_*i*_,*v*_*i*_) and (*X*_*i*_,*Y*_*i*_,*Z*_*i*_) for *i* = 1, 2,…, 8. **S1:** According to *I*_*C*_, 2D coordinates of citrus region extreme points *A*(*u*_*A*_,*v*_*A*_), *B*(*u*_*B*_,*v*_*B*_), *C*(*u*_*C*_,*v*_*C*_), and *D*(*u*_*D*_,*v*_*D*_) are obtained. **S2:** The spatial coordinates of (*X*_*A*_,*Y*_*A*_,*Z*_*A*_), (*X*_*B*_,*Y*_*B*_,*Z*_*B*_), (*X*_*C*_,*Y*_*C*_,*Z*_*C*_), and (*X*_*D*_,*Y*_*D*_,*Z*_*D*_) are calculated by Eqn. (10). **S3:** Citrus fruit diameter*d*_*x*_ and *d*_*y*_ are calculated by Eqn. (11), and *d*_*z*_ is estimated by Eqn. (12). **S4:** According to *I*_*d*_, the spatial coordinates of citrus *Q*_0_(*X*_*q*_,*Y*_*q*_,*Z*_*q*_) are determined by Eqn. (10). **S5:** The distance *d* between *Q*_*0*_ and the origin point in *O*_*c*_ is obtained by Eqn. (13). **S6:** The spatial coordinates (*X*_*i*_,*Y*_*i*_,*Z*_*i*_) of citrus 3D bounding box are calculated by Eqn. (14). **S7:** The 2D coordinates (*u*_*i*_,*v*_*i*_) of citrus 3D bounding box are calculated from (*X*_*i*_,*Y*_*i*_,*Z*_*i*_) using Eqn. (10).

## Results and discussion

The performance of the proposed method was evaluated on a workstation with an Intel Core i9-9920X processor with 3.50 GHz, 32 GB RAM, and an NVIDIA GeForce RTX 2080 GPU with 8 GB RAM. The operating system is Windows 10, and the software framework is PyTorch 1.8. All the algorithms were developed in Visual Studio Code 1.63 and MATLAB R2020a software.

### Performance evaluation of citrus 2D detection

To evaluate the performance of citrus 2D detection using our proposed loss function, (*Loss*_*PB*_), on YOLO v5s, three loss functions, *Loss*_*B*_, focal loss (*Loss*_*F*_) (Lin et al., 2020), and polarity loss (*Loss*_*P*_) (Rahman et al., 2020), were used for comparison. The YOLO v5s models were trained using the training dataset, and the hyperparameters of the model were fine-tuned using the validation dataset. The performance of the final model was evaluated using the test dataset. After several trial-and-error training runs, the learning rate was set to 0.0032, the batch size was set to 32, the IoU threshold was set to 0.5, the training epoch was 200 and γ was set to 20. All the input images were resized to 640 × 640 pixels. The network weights of YOLO v5s were initialized with the weights of the model pretrained on the COCO image dataset.

The detection results under three illumination conditions on the test dataset are provided in Table 2. With our proposed loss function, *Loss*_*PB*_, we achieves the best improvement on the non-uniform illumination than weak illumination and well illumination, compared to *Loss*_*B*_, *Loss*_*F*_, and *Loss*_*P*_. Specifically, under non-uniform illumination, the recall of our loss is 99.55%, which is an average improvement of 9.08% over *Loss*_*B*_, 7.17% over *Loss*_*F*_, and 5.38% over *Loss*_*P*_. The precision of our loss is 95.79%, which is almost the same result as that of the other three loss functions, while the highest precision of 95.93% is obtained by *Loss*_*F*_. The *F*_*1*_-score of our loss is 0.98, which is the highest.

**TABLE 2:** Detection results of You Only Look Once (YOLO) v5s using different loss functions in the test dataset.

Loss function	Illumination	*P* (%)	*R* (%)	*F*_*1*_	*T* (*ms*)	*TP*	*FP*	*FN*
Our Loss *Loss*_*PB*_	Non	95.79	**99.55**	0.98	79.31	888	39	4
	Weak	96.13	98.47	0.97	**75.49**	645	26	10
	Well	**96.64**	98.48	0.98	81.04	1036	36	16
	Total	96.22	98.85	**0.98**	78.96	2569	101	30
*Loss*_*B*_	Non	95.50	90.47	0.93	81.34	807	38	85
	Weak	94.80	91.91	0.93	78.63	602	33	53
	Well	96.07	93.06	0.95	83.16	979	40	73
	Total	95.56	91.88	0.94	81.33	2388	111	211
*Loss*_*F*_	Non	95.93	92.38	0.94	79.91	824	35	68
	Weak	95.09	91.76	0.93	75.38	601	31	54
	Well	96.25	92.78	0.94	82.59	976	38	76
	Total	95.85	92.38	0.94	79.75	2401	104	198
*Loss*_*P*_	Non	95.67	94.17	0.95	79.33	840	38	52
	Weak	95.09	94.66	0.95	75.53	620	32	35
	Well	96.06	95.06	0.96	81.73	1000	41	52
	Total	95.68	94.65	0.95	79.25	2460	111	139

The bold values means the best result on each metrics.

Under weak illumination, the precision of our loss is 96.13%, which is 1.33% higher than that of *Loss*_*B*_ and 1.04% higher than that of *Loss*_*F*_ and *Loss*_*P*_. The recall of our loss is 98.47%, and the *F*_*1*_-score is 0.97, both of which are better than those of the other loss functions. Under well illumination, the *F*_*1*_-score of our loss is 0.98, an average of 3%, 4%, and 2% higher than that of *Loss*_*B*_, *Loss*_*F*_ and *Loss*_*P*_, respectively. The precision and recall of our loss are 96.64% and 98.48%, respectively, which are both the best highest.

Overall, for our loss, the recall is 98.85%, the precision is 96.22%, and the *F*_*1*_-score is 0.98, on average, under the three illumination conditions, values that are approximately 2–9% higher than those of *Loss*_*B*_, about 1–6% higher than those of *Loss*_*F*_, and approximately 1–4% higher than those of *Loss*_*P*_. In terms of other metrics, the detection time per image (*T*) is similar for all loss functions and is consistent with the requirements of the picking robot (Tu et al., 2020).

**FIGURE 8:**
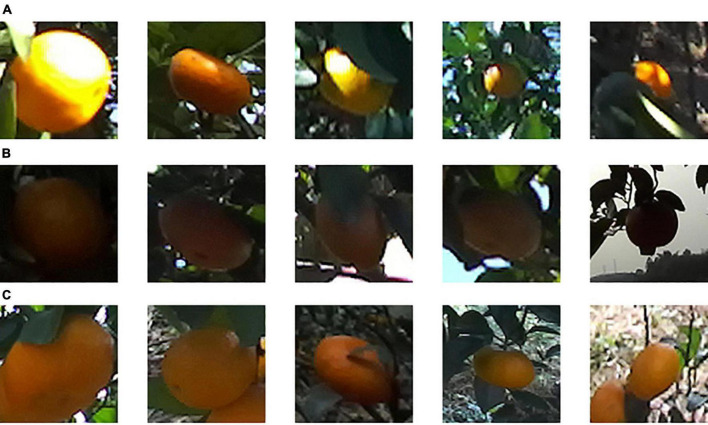
The missed detection of citrus samples of You Only Look Once (YOLO) v5s but detected by our proposed loss in different illumination on test data: **(A)** non, **(B)** weak, and **(C)** well.

Figure 8 shows the citrus samples detected by our loss function *Loss*_*PB*_ but not *Loss*_*B*_ under different illumination conditions. As listed in Table 2, the recall rate of *Loss*_*B*_ under non-uniform illumination is the lowest at 90.47% than other illumination conditions. On the other hand, the recall rate of *Loss*_*PB*_ performed the best at 99.55% over other illumination conditions. The reason may be twofold: (1) As shown in Figure 8, the illumination component is uniform on the surface of a citrus fruit under weak or well illumination conditions. Therefore, the total number of samples is larger under weak and well illumination than under non-uniform illumination, making the YOLO v5s with *Loss*_*B*_ more likely to learn citrus with uniform color features. (2) It is likely that, compared with weak and well illumination, the color features of a citrus fruit under non-uniform will be hard to extract by the Yolo v5s with *Loss*_*B*_, such that the most citrus sample cannot be detected. Using our loss function, the citrus target under non-uniform illumination will be further pushed from the background. A large penalty is applied to missed detection from the penalty function *f*_*P*_ in the training process.

**FIGURE 9:**
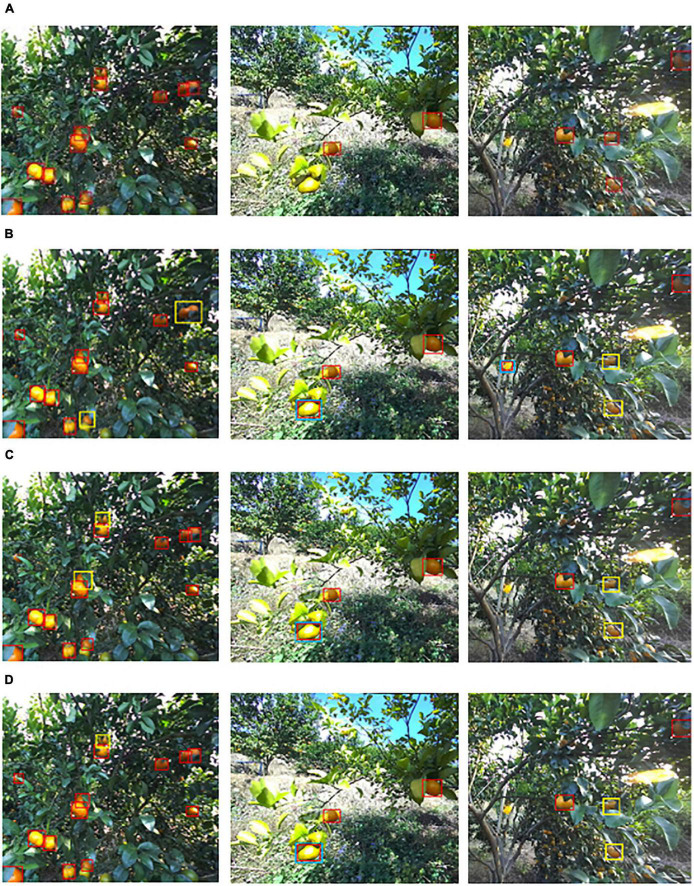
Comparison of detection results using different loss functions: **(A)** Our loss, **(B)** *Loss*_*B*_, **(C)** *Loss*_*F*_, and **(D)** *Loss*_*P*_.

Figure 9 shows the detection results for different loss functions. Specifically, the red bounding box represents the predicted output by models, the yellow bounding box represents the missed detection, and the blue bounding box represents the false detection. Figure 9A indicates that the YOLO v5s model with our loss function achieves the best citrus detection performance under all illumination conditions, reducing the occurrence of both missed detection and false detection.

There are several examples of missed detection or false detection by other loss functions, as presented in Figures 9B–D. With such loss functions, some background objects, such as immature citrus and yellow insect-attracting boards, can lead to false detection of the citrus target. It is likely that immature green citrus has similar texture and shape properties as mature citrus, and the yellow insect-attracting board has similar color characteristics as citrus. On the other hand, citrus that is occluded by leaves, branches, or other backgrounds objects may be misclassified as background, i.e., missed detection. For such citrus fruits, it is likely that only a few features can be extracted from the image, resulting in a hard negative sample that is difficult to distinguish from the background.

Figure 9A shows that our proposed loss function achieves the best detection performance. Specifically, the penalty for false detection is enhanced by the penalty function *f*_*P*_ during the training process, and citrus targets are displaced from the background. As a result, the probability of missed detection is reduced substantially, and the detection performance of citrus is thus improved. Note that *Loss*_*P*_ uses a penalty function similar to *f*_*P*_ and also achieves better performance than that of *Loss*_*F*_ and *Loss*_*B*_. Indeed, it was developed based on *Loss*_*F*_. However, *Loss*_*F*_ cannot push the object further from the background, which may not be an effective improvement on our dataset.

### Performance evaluation of citrus region extraction and depth value restoration

**FIGURE 10:**
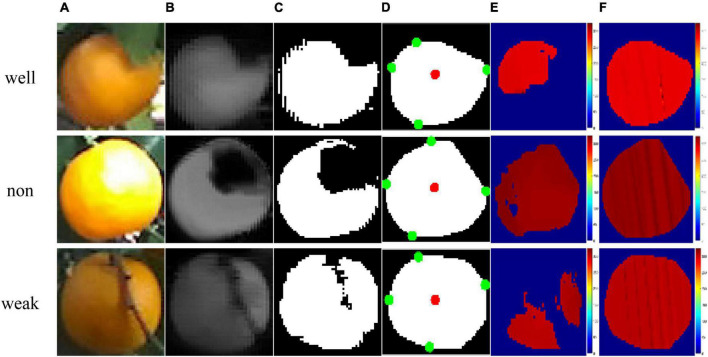
Results of samples under different illumination conditions: **(A)** RGB image, **(B)** Cr-Cb chromatic mapping, **(C)** Otsu segmentation, **(D)** morphological operations, where the red point is the center point and the green point is the maximum and minimum point of the citrus fruit, **(E)** color map of the original depth map, and **(F)** color map on depth map restored by the kriging method.

Figure 10 illustrates the results of citrus region extraction and depth map restoration under variable illumination conditions. Under the well illumination conditions, the citrus occluded by leaves is shown in the first row of Figure 10A. The results of Cr-Cb chromatic mapping and Otsu thresholding are presented in Figures 10B–C. Image noise, holes, and weakly connected regions can exist in the binary image obtained via Otsu thresholding. The citrus region is likely blurred, mainly due to the far distance from the camera. The result of morphological processing is shown in Figure 10D. The image noise was completely removed, and contour smoothing was achieved, such that the majority of the citrus region occluded by the leaves was filled perfectly. As shown in Figure 10E, the depth map of the extracted citrus fruit region after the convex hull operation is incomplete, i.e., the area of missing values covers approximately large than half of the area of the citrus fruit region, which may be caused by camera performance limitations. As shown in Figure 10F, the missing depth values are restored using the kriging method, thereby estimating the complete depth values of the fruit region.

The results of citrus fruit extraction and depth map restoration under the non-uniform illumination conditions are presented in the second row of Figure 10. The shape of the extracted citrus region is obviously incomplete, which may result from overexposure to the citrus surface. As shown in Figure 10D, the incomplete part was restored by morphological operations. Subsequently, the missing depth values in the citrus region (Figure 10E) were recovered, as shown in Figure 10F. Similarly, the results under weak illumination conditions are illustrated in the third row of Figure 10. The citrus fruit region occluded by branches is extracted almost completely, as shown in Figure 10D. Due to the lack of light and other factors, the depth map of the extracted citrus region is sparse, as shown in Figure 10E. After using the kriging method, the missing depth values are effectively restored, as shown in Figure 10F.

**FIGURE 11:**
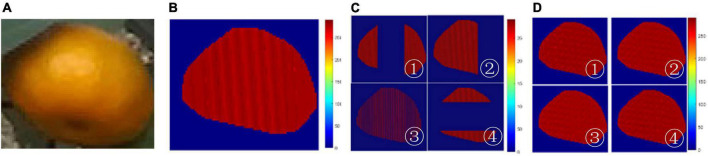
Experiment results using the Kriging method: **(A)** color map of depth values, **(B)** RGB image of a citrus fruit, **(C)** color map by setting depth values zero at random pixels, and **(D)** color map of restoration by kriging.

To evaluate the accuracy of the kriging method to recover depth values on the occluded citrus region, an experiment was conducted by simulating the restoration using the incomplete depth map. Figure 11 shows the results of using the kriging method on an extracted citrus region. Figure 11A is the complete depth map of Figure 11B. Figure 11C shows that the incomplete depth map was generated by setting the corresponding depth values to zero with four schemes. About 50% of the pixels are set as missing values. Specifically, the incomplete depth maps ① and ④ were created by setting the pixels of the central part to zero in the vertical and horizontal directions. The incomplete depth map ② was created by setting the pixels of the right part to zero, and ③ was created by setting the interleaving pixels to zero. As shown in Figure 11D, the missing values are recovered using the kriging method, such that the depth map of the fruit region is completely restored.

Compared with the original depth map of Figure 11B, the average restoration error of depth map ①, ②, ③, and ④ is 2.29, 2.15, 2.08, and 2.31 mm, respectively, such that the average of the all the restoration errors is 2.21 mm. The minimum error was performed in the depth map ③, indicating that the estimate of missing depth value is recovered with high accuracy when the depth values are only missing randomly in the depth map. On the other hand, the maximum error was performed in the depth map ① and ④, indicating that the restoration error is large when the missing depth values are in the most discontinuous part of the depth map. In total, the mean relative error is 1.36%, indicating that the kriging method effectively restored the depth map with high accuracy.

### Performance evaluation of citrus 3D localization

Citrus diameter *d*_*x*_, *d*_*y*_, and *d*_*z*_, coordinates of citrus *Q*_0_(*X*_*q*_,*Y*_*q*_,*Z*_*q*_), the distance between the citrus and camera *d*, the 3D coordinates of the citrus 3D bounding box (*X*_*i*_,*Y*_*i*_,*Z*_*i*_), and its 2D coordinates (*u*_*i*_,*v*_*i*_) are calculated using Algorithm 1. Specifically, to obtain the regression model for *d*_*z*_, as mentioned in Eqn. (12), a total of 137 citrus samples were collected in the orchard. The diameter *d*_*x*_, *d*_*y*_, and *d*_*z*_ of each fruit were measured by a Vernier caliper (Pro skit, PD-151). The quadratic polynomial function fitted for *d*_*z*_ is determined as follows:


(15) $${\hat{d}}_{z}=16.0728+0.0028\,d_{x}^{2}+0.0018\,d_{y}^{2}+0.0264\,d_{x}+0.4133\,d_{y},$$

where the root mean square error (RMSE) is 4.51 mm and the coefficient of determination *R*^2^ = 0.940, indicating a good model for estimating *d*_*z*_.

**FIGURE 12:**
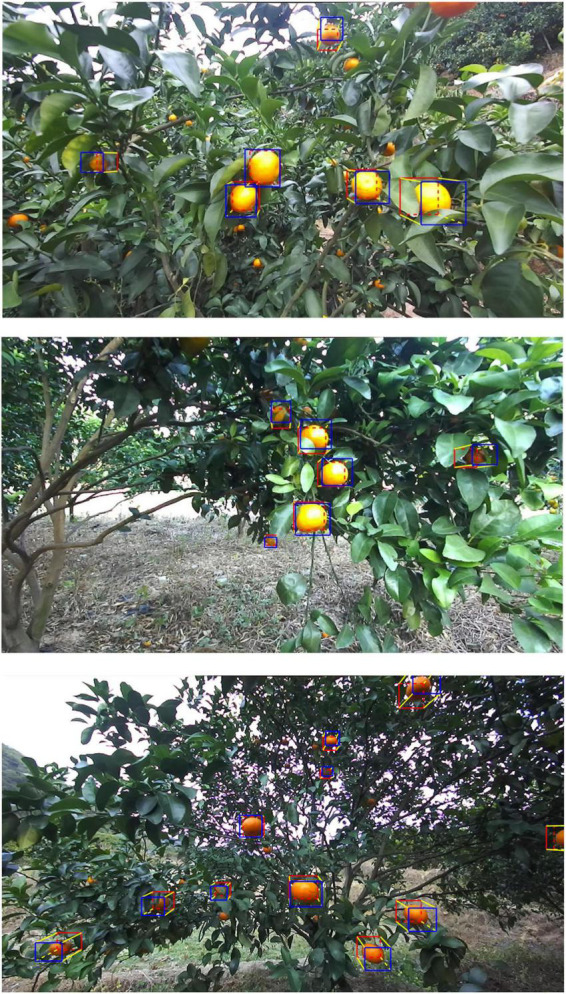
Examples of 3D bounding boxes for citrus fruits.

Figure 12 shows the result of 3D bounding boxes predicted for each citrus fruit. The boxes are drawn by connecting the adjacent vertices (*u*_*i*_,*v*_*i*_), for *i* = 1, 2,…, 8, with a straight line. The front face of the 3D bounding box was drawn by the blue rectangle, the back face of the 3D bounding box was drawn by the red rectangle, and the side face of the 3D bounding box was drawn by the yellow line. The citrus fruits near the center of the image are correctly detected with the 3D bounding boxes. Moreover, the four edge lines (yellow lines) of the 3D bounding box disappear in the center of the image, which is consistent with the principle of parallel perspective (Cai et al., 2021). Thus, our proposed method achieves accurate localization results.

**FIGURE 13:**
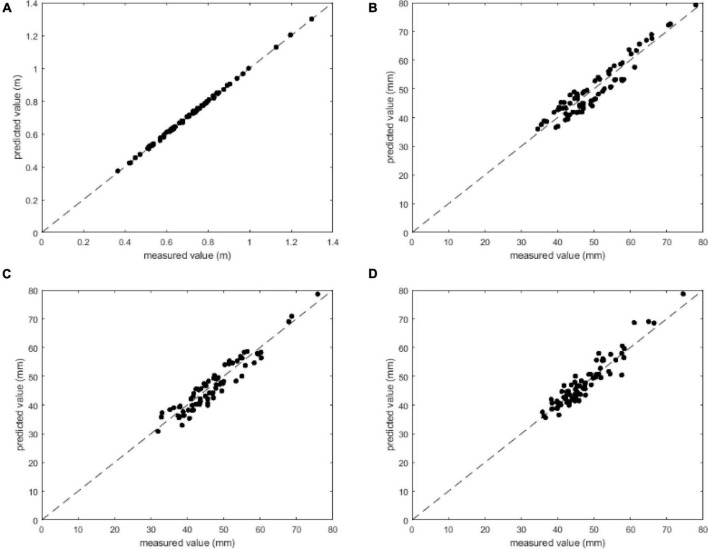
Comparison between the measured values and predicted values: **(A)** *d*, **(B)** *d*_*x*_, **(C)** *d*_*y*_, and **(D)** *d*_*z*_.

To evaluate the localization accuracy of citrus 3D localization, 22 images of citrus fruits were considered. The distance between the citrus and the camera *d* was measured by a laser rangefinder (UNI-T, UT392B), and citrus diameters *d*_*x*_, *d*_*y*_, and *d*_*z*_ were measured with a Vernier caliper (Pro skit, PD-151). A scatter plot of the measured values and the values predicted by our method is presented in Figure 13. Our method obtains good accuracy for predicting *d*, *d*_*x*_, *d*_*y*_, and *d*_*z*_: the closer the measured values and the predicted value are to the 45-degree line, the higher the accuracy. Figure 13A shows the best prediction and fewer errors between the measured value and predicted values for *d*, where all the plotted points lie almost on the 45-degree line. Furthermore, Figures 13B–D shows that the predicted values of *d*_*x*_, *d*_*y*_, and *d*_*z*_ are generally close to the 45-degree line, indicating that our proposed method is able to achieve accurate localization results.

Overall, the average error of distance *d* between the citrus and camera is 3.98 mm, which is better than the 15 mm achieved in Wang et al. (2016). The average errors of citrus diameters *d*_*x*_, *d*_*y*_, and *d*_*z*_ were 2.75, 2.52, and 2.11 mm, respectively, which is almost the same precision as (Yang et al., 2020) and better than the 10 mm achieved in (Nguyen et al., 2016) and the 4.9 mm achieved in (Wang et al., 2017).

Our method can accurately locate citrus under variable illumination and different occlusion conditions in natural orchards. The distance *d* can be used to determine the extension length of the robot hand, and the coordinates of citrus *Q*_0_(*X*_*q*_,*Y*_*q*_,*Z*_*q*_) can be used to manipulate the robot hand’s the series of joints or articulations. The diameter *d*_*x*_, *d*_*y*_, *d*_*z*_ and the 3D bounding box (*X*_*i*_,*Y*_*i*_,*Z*_*i*_) can be used to finetune the posture of grasping structures.

## Conclusion

This paper aims to address the problem of the lower detection rate for mature citrus under variable illumination and occlusion conditions. We proposed a novel method to detect and localize citrus fruits in natural orchards using binocular cameras and deep learning. The main conclusions are as follows:

1. A new loss function *Loss*_*PB*_ for YOLO v5s is proposed to calculate the loss value for class probability and objectness score, with a penalty function *f*_*p*_ developed to account for the disparity between citrus and background. As a result, the citrus detection performance of our loss function is improved by pushing the citrus further from the background in the training process, even under variable illumination and different occlusion conditions. The recall values of the three groups of illumination conditions were 99.55%, 98.47%, and 98.48%, the precision values were 95.79%, 96.13%, and 96.64%, respectively, and the *F*_*1*_-scores were close to 0.98. The average detection time was 78.97 ms per image. Compared with the original YOLO v5s, the performance improvement was 2-9% on average.
2. Based on the detected 2D bounding box for a citrus, the potential fruit region of mature citrus was segmented completely using Cr-Cb chromatic mapping, Otsu thresholding and morphology processing. In particular, the difference in color intensity between citrus targets and background objects is enhanced using Cr-Cb chromatic mapping, which helps to extract the complete shape of citrus fruit using Otsu thresholding and morphology processing.
3. To recover the missing depth values in the citrus region under different occlusion states, the kriging method was applied based on the spatial proximity among neighboring points. The experimental results show that the average error of the restored depth values was 2.02 mm and the relative error was 1.26%, indicating that the method can accurately restore the depth map of citrus fruit.
4. Based on the ellipsoid characteristic of citrus fruit, the 3D localization information of citrus is accurately determined using the camera imaging model and a restored depth map. The experimental results show that the average error of the distance *d* between the citrus fruit and the camera was 3.98 mm, and the average errors of the citrus diameter *d*_*x*_, *d*_*y*_ and *d*_*z*_ were 2.75, 2.52, and 2.11 mm, respectively, which is better than the results achieved in other research.

Our method can provide 3D citrus position data under variable illumination and different occlusion conditions in natural orchards. Future work will focus on few-shot learning and reduce the number of citrus fruits in the training dataset to improve citrus detection and localization.

## Data availability statement

The original contributions presented in this study are publicly available. This data can be found here: https://github.com/AshesBen/citrus-detection-localization.

## Author contributions

All authors contributed to the method and result of the study, dataset generation, model training and testing, analysis of results, and the drafting, revising, and approving of the contents of the manuscript.
